# Cytogenetics, genomics and biodiversity of the South American and African Arapaimidae fish family (Teleostei, Osteoglossiformes)

**DOI:** 10.1371/journal.pone.0214225

**Published:** 2019-03-25

**Authors:** Ezequiel Aguiar de Oliveira, Luiz Antonio Carlos Bertollo, Petr Rab, Tariq Ezaz, Cassia Fernanda Yano, Terumi Hatanaka, Oladele Ilesanmi Jegede, Alongklod Tanomtong, Thomas Liehr, Alexandr Sember, Sandra Regina Maruyama, Eliana Feldberg, Patrik Ferreira Viana, Marcelo de Bello Cioffi

**Affiliations:** 1 Departamento de Genética e Evolução, Universidade Federal de São Carlos (UFSCar), Rodovia Washington Luiz, São Carlos, SP, Brazil; 2 Secretaria de Estado de Educação de Mato Grosso–SEDUC-MT, Cuiabá, MT, Brazil; 3 Laboratory of Fish Genetics, Institute of Animal Physiology and Genetics, Czech Academy of Sciences, Czech Republic; 4 Institute for Applied Ecology, University of Canberra, Canberra, Australia; 5 Department of Fisheries and Aquaculture, Adamawa State University, Adamawa State, Nigeria; 6 Toxic Substances in Livestock and Aquatic Animals Research Group, KhonKaen University, Muang, KhonKaen, Thailand; 7 Institute of Human Genetics, University Hospital Jena, Jena, Germany; 8 Instituto Nacional de Pesquisas da Amazônia, Coordenação de Biodiversidade, Laboratório de Genética Animal, Petrópolis, CEP: Manaus, AM, Brazil; SOUTHWEST UNIVERSITY, CHINA

## Abstract

Osteoglossiformes represents one of the most ancestral teleost lineages, currently widespread over almost all continents, except for Antarctica. However, data involving advanced molecular cytogenetics or comparative genomics are yet largely limited for this fish group. Therefore, the present investigations focus on the osteoglossiform family Arapaimidae, studying a unique fish model group with advanced molecular cytogenetic genomic tools. The aim is to better explore and clarify certain events and factors that had impact on evolutionary history of this fish group. For that, both South American and African representatives of Arapaimidae, namely *Arapaima gigas* and *Heterotis niloticus*, were examined. Both species differed markedly by diploid chromosome numbers, with 2n = 56 found in *A*. *gigas* and 2n = 40 exhibited by *H*. *niloticus*. Conventional cytogenetics along with fluorescence *in situ* hybridization revealed some general trends shared by most osteoglossiform species analyzed thus far, such as the presence of only one chromosome pair bearing 18S and 5S rDNA sites and karyotypes dominated by acrocentric chromosomes, resembling thus the patterns of hypothetical ancestral teleost karyotype. Furthermore, the genomes of *A*. *gigas* and *H*. *niloticus* display remarkable divergence in terms of repetitive DNA content and distribution, as revealed by comparative genomic hybridization (CGH). On the other hand, genomic diversity of single copy sequences studied through principal component analyses (PCA) based on SNP alleles genotyped by the DArT seq procedure demonstrated a very low genetic distance between the South American and African Arapaimidae species; this pattern contrasts sharply with the scenario found in other osteoglossiform species. Underlying evolutionary mechanisms potentially explaining the obtained data have been suggested and discussed.

## Introduction

Freshwater fishes represent an important model group for biogeographic studies, as their evolution is often tightly linked with (and affected by) the Earth's geological development that shapes the freshwater networks [1,2]. Consequently, each continent presents characteristic freshwater fish faunas, where the proper distributional patterns are modified by physical barriers that blocked the dispersion of ancestors for many present-day species. The freshwater order Osteoglossiformes is an important model for biogeographic studies [3–5] as it represents one of the main ancestral teleostean lineages [6–8] that shows a worldwide distribution, with at least one representative in each continent of the southern hemisphere, except for Antarctica. According to the current taxonomy, Osteoglossiformes includes the suborders Osteoglossoidei (including the Osteoglossidae, Arapaimidae, Pantodontidae families) and Notopteroidei (including the Gymnarchidae, Mormyridae, and Notopteridae families) [6,9].

**Fig 1:**
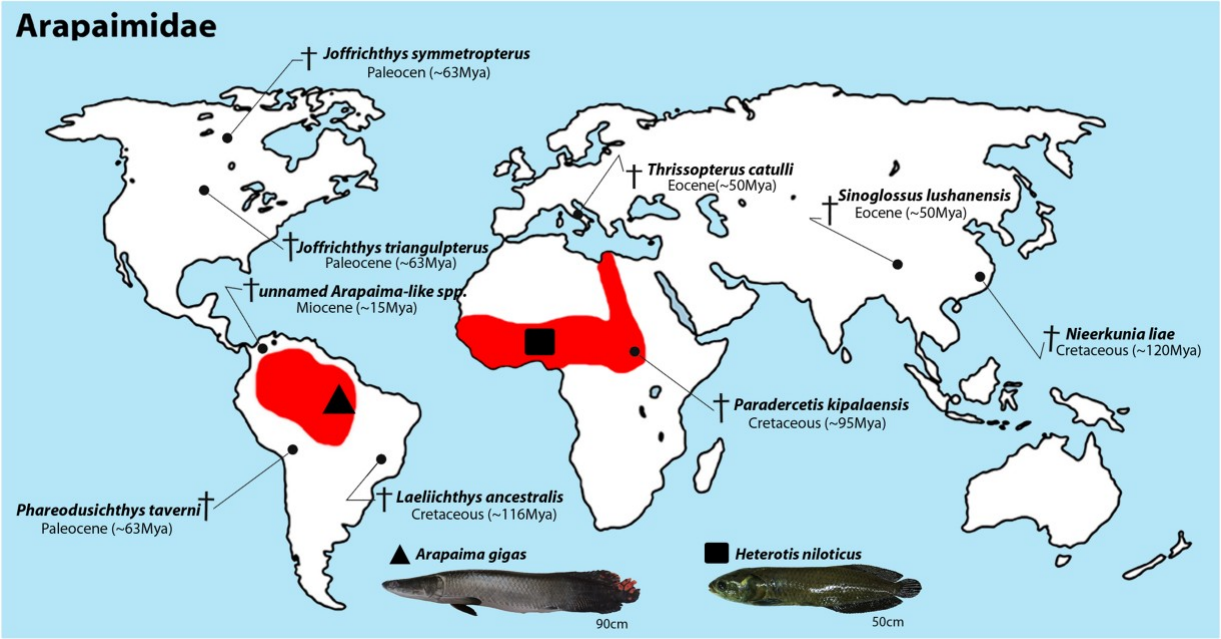
Geographical distribution of Arapaimidae fishes and the sampling locations. Distribution areas of extant Arapaimidae species (red) and the currently described fossil records (crosses). The fossil records are based on descriptions reported by [4,9,22–27]. The sampling sites are marked by triangle for *Arapaima gigas* (Brazil) and square for *Heterotis niloticus* (Nigeria).

Arapaimidae includes the South American genus *Arapaima* and the African genus *Heterotis*, which are distributed in various freshwaters of these continents (Fig 1). *Heterotis* is reputedly represented by the sole bonytongue species *H*. *niloticus*, but the actual species diversity is unknown, with some preliminary reports indicating possible fast ongoing genetic differentiation [10,11]. The African bonytongue can reach up to 1 m in length, and, due to the demand and popularity of their meat [10], this species has experienced a huge population decrease and has been recently included in the list of endangered species [12].

*Arapaima* has been considered monotypic since Günther grouped *A*. *arapaima*, *A*. *mapae*, and *A*. *agassizii* into a single taxon, *A*. *gigas* [13–15]. However, recent studies have led to the conclusion that all three taxa are valid as separate, well-diagnosed species and one additional new species, *A*. *leptosoma*, has been described, with further indications that the list of *Arapaima*´s species will likely continue to grow [14,15]. Hence, *Arapaima* genus lost the monotypic status, encompassing more species than appreciated for more than one century. The real problem may be represented by the fact that these species are known just from their holotypes and their actual distribution is unknown, because all *Arapaima* individuals are recognized as *A*. *gigas*, popularly known as “pirarucus”. The natural distribution of these fishes covers a large part of the Brazilian Araguaia–Tocantins and Amazon River basins [16,17]. They live preferentially in lentic environments such as floodplains and lakes, with significant sedentary behavior [18,19], displaying complex reproductive strategies, including nest building and a high level of parental care [20, 21]. Being one of the largest freshwater fishes of the planet, with individuals measuring up to 4 m in length, they have been subjected to extensive fishery activities for years [6].

The break-up of Gondwana has been discussed as being the driving force for the speciation and intercontinental distribution of the extant arapaimids. However, based on the fossil record, common ancestors of living Arapaimidae were widespread in the world, inhabiting both Laurasia and Gondwanaland (Fig 1). This is also consistent with molecular evidences suggesting that the diversification into osteoglossiform (sub-) families started before the Pangea break-up [3,5]. Extant living arapaimids are represented by only two genera, namely the African *Heterotis* which is native to all basins of the Sahelo-Sudanese region, Senegal, Gambia, Corubal, Volta, Ouémé, Niger, Bénoue, Chad and Nile basins, and the South American *Arapaima*, widely distributed throughout the Amazon lowlands, Araguaia-Tocantins river basin and Guiana. Consequently, an intriguing question arises: how do the living arapaimids reach the South American territory? In this sense, speculations on the role of vicarious events in the divergent process among such species are plausible. However, while geological data suggest that Africa and South America started to separate in the early Cretaceous (~135 Mya) [28], slowly settling in the middle of the Cretaceous between 120–110 Mya with the increase of the Atlantic Ocean to the south [29], by using nuclear and mitogenomic markers and calibration with fossil records, it was estimated that the time of divergence between these two lineages occurred within the range of 85 to 50 Mya [5], thus requiring alternative biogeographic hypotheses to explain the current intercontinental distribution of these fishes. Therefore, diversified approaches, including karyotype and high-resolution sequencing analyzes have been employed and interpreted within the context of Arapaimidae biogeographic history, in order to provide novel complex insight into this matter.

Advances in cytogenetic techniques have provided evidence that repetitive DNA sequences play an important role in the structural and functional organization of the genomes [30,31]. In the last two decades, large amounts of data were generated concerning the chromosomal mapping of repetitive DNAs in several fish species [32]. However, the cytogenetic data for representatives of Osteoglossiformes are still scarce and, when available, they originate from studies performed in the early 1970s and are mainly restricted to the description of the diploid number (2n) and the karyotype structure. A summary table with such information can be found in Ráb et al. [33]. Within Arapaimidae, only two studies reporting the distribution of the constitutive heterochromatin and the mapping of rDNA sequences in chromosomes have been performed up to now [34,35].

Comparative genomic hybridization (CGH) is a FISH-based method that utilize total genomic DNAs (gDNAs) of the investigated organisms as a hybridization probe. Through CGH, it is possible to compare the genomic content from two (or more) different sources on the level of gross molecular composition, once the probes are simultaneously hybridized onto chromosomal preparations of interest [36–39]. The principle of the method is based on the differential distribution of already divergent genome-specific repetitive DNA classes, as this highly abundant genomic fraction display faster evolutionary rate than the single-copy regions [31,40,41]. Such methodology has been, for instance, successfully applied for i) the identification of parental genomes in hybrids/allopolyploids, ii) delimitation of sex-specific regions on both homomorphic and heteromorphic sex chromosomes or iii) the genome comparisons among related species (for references, see [42]).

Here we performed comprehensive molecular cytogenetic analysis for the representatives of Arapaimidae family by applying differential cytogenetic methods, such as C-banding, CGH and physical mapping of certain repetitive DNA classes through FISH, complemented with DArT-seq molecular analysis using single nucleotide polymorphisms (SNPs). The main goal was to investigate and compare patterns of the chromosomal and the gross-scale genome evolution in two representatives of Arapaimidae from two different continents and to interpret them within the context of known data from other osteoglossiform fishes, bringing thus new insights into evolutionary trends within the group.

## Materials and methods

### Animals, mitotic chromosome preparation, and banding procedures

Individuals unambiguously identified as *Heterotis niloticus* (four females and four males; Oluwa River (Africa), 6°16'60.0"N 4°49'00.0"E) and *Arapaima gigas* (seven females and eight males; Tocantins River basin (South America), 11°5'14.90"S, 49°56'21.72"W) were analyzed (Fig 1).

All the specimens of *Heterotis niloticus* were deposited under voucher number 20558 in the Museum of Universidade Estadual Paulista (UNESP, Botucatu). The specimens of *Arapaima gigas* were deposited in the Museum of Zoology of the University of São Paulo (MZUSP), under voucher number 121639. Samples were collected with the authorization of the Brazilian environmental agency ICMBIO/SISBIO (nº 48290–1) and SISGEN (n^o^ A96FF09). No authorization for sampling the African specimens was required. The identity of *A*. *gigas* was determined based on diagnostic characters provided by Stewart [14,15]. In order to increase the number of mitotic cells, animals were first stimulated with yeast suspensions for 48 h [43]. Next, they were euthanized with an overdose of benzocaine (1 g/L) and sacrificed for chromosome preparation, which was done following Bertollo et al. [44], with few modifications, but with one major necessary improvement in the way that we used cells derived from the spleen instead of kidney to obtain mitotic chromosomes of sufficient quality (full protocol details are available as **S1 Appendix.** The experiments were approved by the Ethics Committee on Animal Experimentation of the Universidade Federal de São Carlos (Process in CEUA 1926260315).

C-banding, silver-nitrate impregnation (Ag-NOR) and Chromomycin A_3_ (CMA_3_) staining were performed following protocols described by Howell and Black [45], Schmid [46] and Sumner [47], respectively.

### Probe preparation and fluorescence *in situ* hybridization (FISH) analysis

5S and 18S rDNA fragments were obtained by polymerase chain reaction (PCR) using primers and thermal profiles described in Martins et al. [48] and Cioffi et al. [49], respectively. The 5S rDNA probe was composed of 120 base pairs (bp) of the 5S rRNA-encoding gene and 200 bp of the non-transcribed spacer (NTS), while the 18S rRNA probe encompassed a 1400 bp long segment corresponding to the 18S rRNA gene. The 18S rDNA probe was labeled with Spectrum Orange-dUTP (Vysis, Downers Grove, IL, USA) while the 5S rDNA probe was labeled with Spectrum Green-dUTP (Vysis, Downers Grove, IL, USA), both by nick translation kit, according to the manufacturer’s recommendations (Roche, Mannheim, Germany).

Fluorescence *in situ* hybridization (FISH) was performed following Pinkel et al. [50]. The chromosome preparations were incubated with RNase (40 μg/mL) for 1.5 h at 37°C. After denaturation of the chromosomal DNA for 3min in 70% formamide/2× SSC at 70°C, spreads were dehydrated in an ethanol series (70, 85, and 100%), 2 min each. Then, 20 μL of the hybridization mixture (100 ng of each probe, 50% deionized formamide, 10% dextran sulphate) was dropped onto the slides, and the hybridization was performed for 14 h at 37°C in a moist chamber containing 2× SSC. The post-hybridization wash was carried out with 1× SSC for 5 min at 42°C. A final wash was performed at room temperature in 4× SSC for 5 min. Finally, the chromosomes were counterstained with DAPI (1.2 μg/mL) and mounted in antifade solution (Vector, Burlingame, CA, USA).

### Comparative genomic hybridization (CGH)

The CGH experiment was performed according to Symonová et al. [39]. For each probe, 1 μg of gDNA was used in the labeling procedure. *A*. *gigas* gDNA was labeled with digoxigenin-11-dUTP using DIG-nick-translation Mix (Roche), while the *H*. *niloticus* gDNA was labeled with biotin-16-dUTP using BIO-nick-translation Mix (Roche). The hybridization solution for each slide (25 μL) was composed of 1 μg of each genomic probe and 50 μg of unlabeled C_0_t-1 DNA (i.e. fraction of genomic DNA enriched for highly and moderately repetitive sequences). C_0_t-1 DNA was directly isolated from both species according to Zwick et al. [51]. The chosen ratio of probe vs. C_0_t-1 DNA amount was set based on the experiences gained during analogous experiments performed in our previous studies in fishes [42,52–57]. The chosen ratio 1:50 reflects high stringency towards repetitive DNA blocking and yet avoids the probability of improper probe dissolution in the hybridization buffer, which would otherwise cause artifacts [39,42].

Chromosome preparations were stored overnight in a freezer; they were passed through an ethanol row (70, 85, and 100%, 3 min each) before and after the storage. After that, the slides were aged for 1–2 h at 60°C and treated gradually with RNase (200 μg/mL, 90 min at 37°C in a wet chamber) and with pepsin (50 μg/mL in 10 mM HCl, 3 min, 37°C). Finally, chromosomes were denatured in 75% formamide/2× SSC at 72°C for 5 min, and immediately passed through 70% (cold), 85%, and 100% (Room Temperature) ethanol series (3 min each). The probe mixture (20 μL) was first denatured at at 86°C for 8 min and then applied onto the slides, which were then incubated at 37°C in a dark humid chamber for 72 h. The slides were then washed twice in 50% formamide/2× SSC for 10 min each and incubated with 500 μL of 3% bovine serum albumin (BSA)/4× SSC/Tween (20 min, 37°C). The hybridization signals were detected with anti-Digoxigenin-Rhodamine (Roche) diluted in 0.5% BSA in PBS, and avidin-FITC (Sigma) diluted in PBS containing 10% normal goat serum (NGS). The final washes were performed at 44°C in 4× SSC and 0.01% Tween: three washes, 7 min each. Finally, the chromosomes were counterstained with DAPI as described above.

### Microscopic analyses and image processing

At least 30 metaphase spreads per individual were analyzed to confirm the 2n number, karyotype structure, and results of FISH experiments. Images were captured using an Olympus BX50 microscope (Olympus Corporation, Ishikawa, Japan) with CoolSNAP, and the images were processed using Image Pro Plus 4.1 software (Media Cybernetics, Silver Spring, MD, USA). Chromosomes were classified as metacentric (m), submetacentric (sm), subtelocentric (st) and acrocentric (a) based on Levan et al. [58].

### DNA extraction and DArT-seq analysis

Liver tissue was obtained and stored in 100% ethanol for DNA extraction (for protocol details, see [59]). Besides *H*. *niloticus* and *A*. *gigas*, DNA from other Notopteridae species, namely *Chitala blanci*, *C*. *ornata*, *C*. *lopis*, *Notopterus notopterus*, *Xenomystus nigri* and *Papyrocranus afer*, was also extracted and used for DArT-seq analysis [60]. The gDNAs were analysed under the DArT-seq technology [61] by the Diversity Arrays Technology Company (Canberra, Australia). A combination of PstI and SphI enzymes was used to construct the libraries using methods described by [62], and sequenced on the Illumina Hiseq2500 next generation sequencer. These enzymes were selected since both are 6 base cutter targeting AG and GC rich regions and thus they indirectly target gene rich regions of the genome. Two libraries were constructed for each DNA sample and the whole process of data generation was done in full technical replication (from digestion/ligation step to marker calling). Approximately 2.5 million sequences were used per sample to produce marker data. Markers were extracted using DArT PL’s proprietary analytical pipeline which, in addition to allele calling and marker data metadata reporting, evaluates consistency of allele calling among the technical replicates.

Single-nucleotide polymorphisms (SNPs) and SilicoDArTs markers were extracted from the sequences of genomic representations (libraries). SilicoDArTs, which represent presence/absence of specific restriction fragment in genomic representations were scored as “1” for the “present” allele and “0” for absence of the fragment/sequence. SNPs were scored in “two row” format–each row representing a specific allele at the SNP locus. The absence of the allele was scored ‘0’ and ‘1’ was reported for presence of the allele **(see S1 Table for details)** [62].

### Analysis of genetic diversity between species

From the filtered SNP DArT-seq data matrix, a pair-wise genetic similarity matrix, based on [63], was computed and utilized for the genetic diversity analyses through R packages. The principal component analysis (PCA) was performed with FactorMineR [64], while hierarchical clustering analysis with *p-*values (AU, Approximately Unbiased *p*-value and BP, Bootstrap Probability value) was performed with pvclust [65] using Euclidean distance.

## Results

### Karyotype analysis and distribution of constitutive heterochromatin

**Fig 2:**
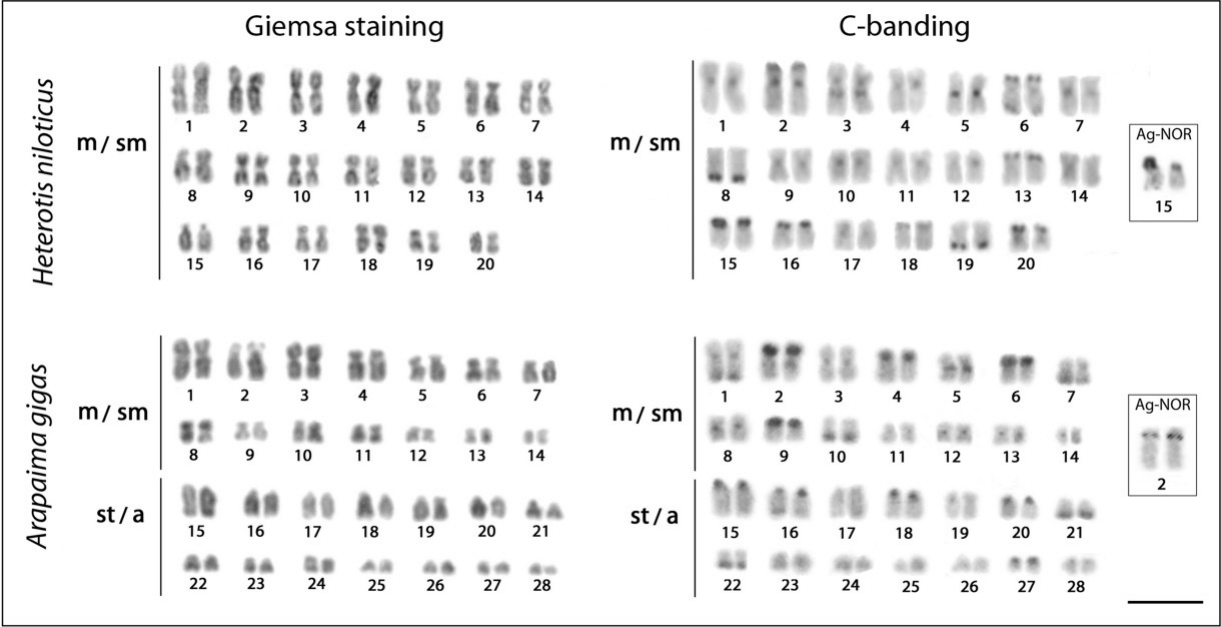
Karyotypes of *Arapaima gigas* and *Heterotis niloticus* arranged from Giemsa-stained and C-banded chromosomes. The Ag-NOR pairs are highlighted in boxes. Bar = 5 μm.

Both species displayed identical karyotypes for males and females, without cytologically detectable sex-related heteromorphisms. Individuals of *A*. *gigas* exhibited 2n = 56, with karyotype composed of 28 metacentric (m) to submetacentric (sm) and 28 subtelocentric (st) to acrocentric (a) chromosomes, and with a number of chromosomal arms per cell (FN; Fundamental number) being equal to 84. On the other hand, karyotype of *H*. *niloticus* consisted of 2n = 40, with all chromosomes possessing bi-armed (i.e. metacentric or submetacentric) morphology and FN = 80. In both species, the C-positive bands of constitutive heterochromatin were found to reside preferentially in the centromeric/pericentromeric regions of all chromosomes, with some additional conspicuous telomeric blocks being present in a subset of chromosome pairs. Specifically, conspicuous terminal and interstitial segments of constitutive heterochromatin were observed in pairs 2, 3, 6, 8, 13, 15, 16, 18, 19, and 20 in *H*. *niloticus* and pairs 1, 2, 4, 6, 7, 9, 10, 21, and 22 in *A*. *gigas*, with the bands on pairs 15 (*H*. *niloticus*) and 2 (*A*. *gigas*) corresponding to locations of NORs as revealed by silver-nitrate staining (see Fig 2).

### Patterns of 5S/18S rDNA and CMA_3_-positive sites distribution

**Fig 3:**
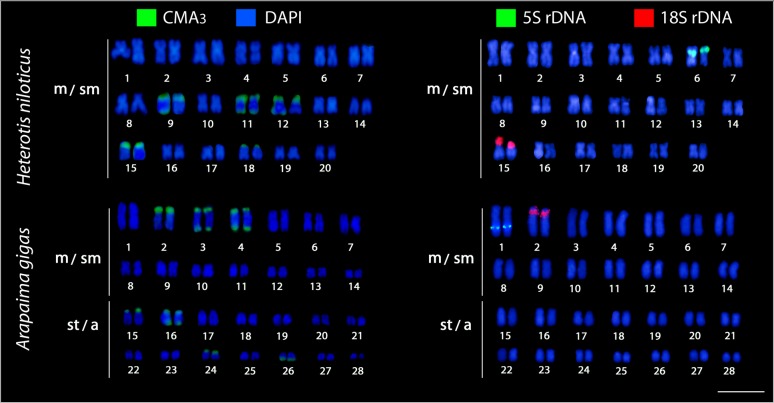
Karyotypes of *Arapaima gigas* and *Heterotis niloticus* after Chromomycin A_3_/DAPI-staining and rDNA FISH. Dual-colour FISH was performed using 18S (red) and 5S (green) rDNAs as probes. Bar = 5μm.

FISH with the 5S rDNA probe showsbright signals in the pericentromeric region of chromosome pair 6 in *H*. *niloticus*, whereas in *A*. *gigas*, these signals were placed interstitially on the *q* arms of chromosome pair 1. The 18S rDNA probe identified a single locus with a very intense signal located in the distal *p* arm of pair 15 in *H*. *niloticus* and in the proximal *p* arm of pair 2 in *A*. *gigas*, corresponding to the Ag-NOR sites in both species (Fig 3).

Fluorescence staining detected multiple CMA_3_-positive bands in the genomes of both species, particularly nearby the telomeric region of five chromosome pairs in *H*. *niloticus* (chromosomes 9, 11, 12, 15, and 18) and of seven chromosome pairs in *A*. *gigas* (chromosomes 2, 3, 4, 15, 16, 24, and 26), in addition to CMA_3_-positive Ag-NOR sites (Fig 3).

### Patterns of cross-specific CGH

**Fig 4:**
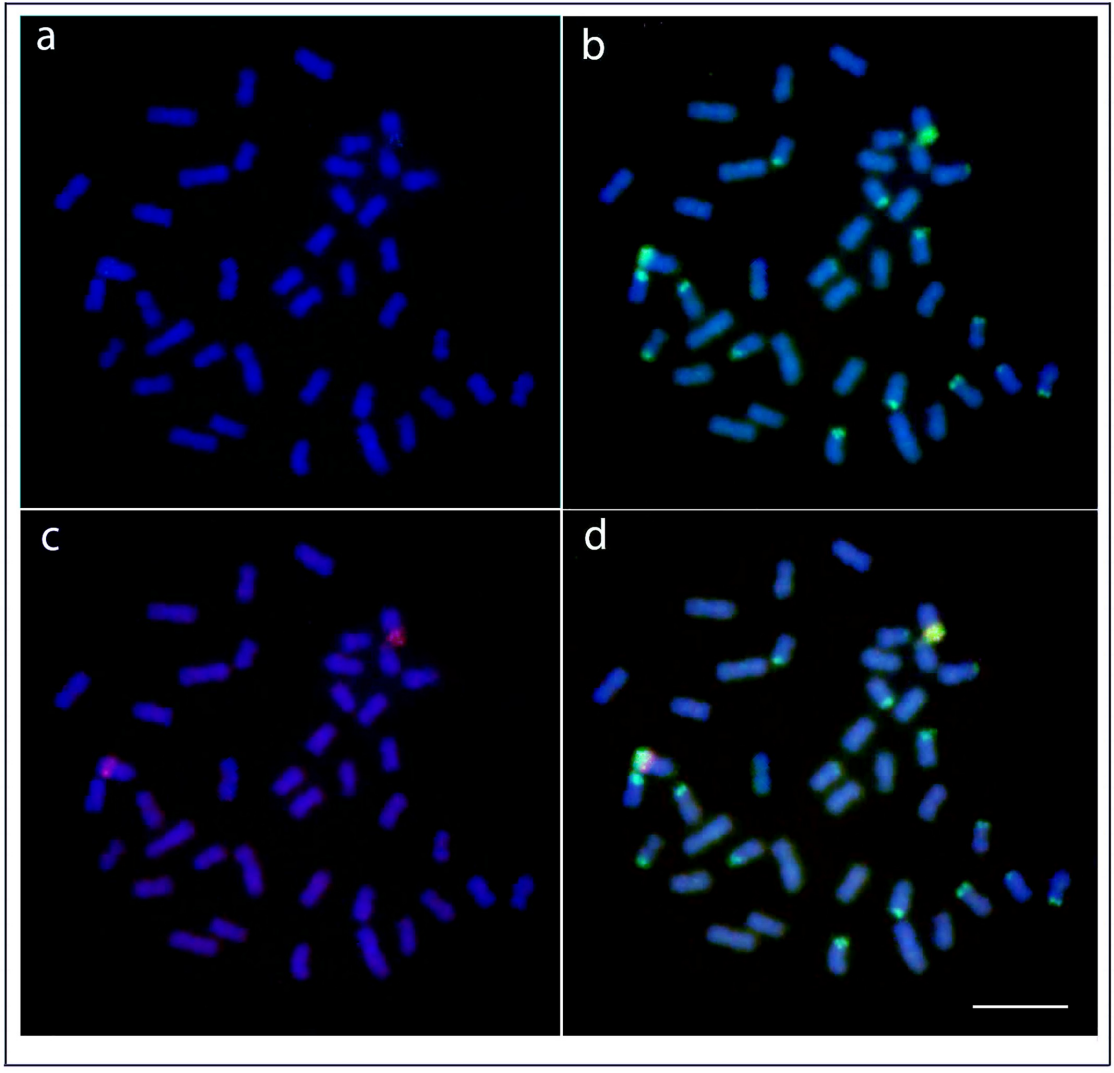
Comparative genomic hybridization (CGH) on metaphase chromosomes of *Heterotis niloticus*. (A) DAPI-stained chromosomes(B) Hybridization pattern with probe derived from gDNA of *Heterotis niloticus* (green); (C) Hybridization pattern with probe derived from with gDNA of *Arapaima gigas* (red); (D) Superposition of both gDNA probes showing the shared sequences between the species. Chromosomes were counterstained with DAPI (blue). Bar = 5 μm.

**Fig 5:**
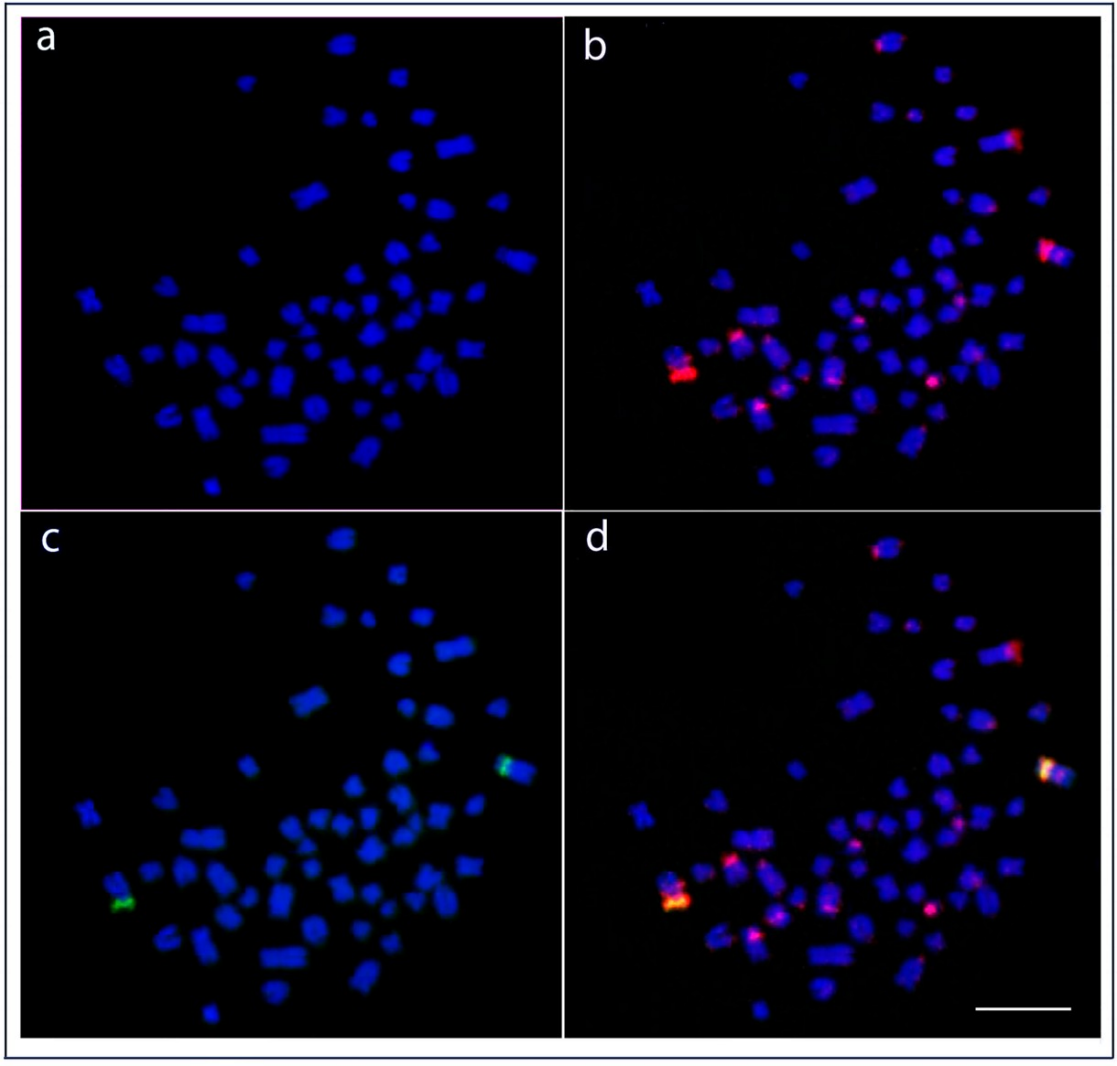
Comparative genomic hybridization (CGH) on metaphase chromosomes of *Arapaima gigas*. (A) DAPI-stained chromosomes (B) Hybridization pattern with probe derived from gDNA of *Arapaima gigas* (red); (C) Hybridization pattern with probe derived from gDNA of *Heterotis niloticus* (green); (D) Superposition of both gDNA probes showing the shared sequences between the species. Chromosomes were counterstained with DAPI (blue). Bar = 5 μm.

The CGH experiments provided information about major differences between analyzed genomes regarding amount and distribution of the shared vs. genome-specific repetitive DNA fraction. As expected, both genomes shared only minor portion of repetitive DNA sequences, specifically only a segment related to CMA_3_-positive/NOR/18S rDNA regions (showed as yellow signals, i.e. combination of green and red). Additionally, in both experimental designs, the probe derived from the gDNA of the species whose chromosomes are subjected to analysis (i.e. hybridization back against its own chromosome complement) hybridized preferentially to heterochromatic blocks abundantly present in the terminal chromosomal regions (as evidenced by sequential C-banding analysis), despite the high amount of competitive DNA. It should be, however, noted that despite less intensely, the conspecific genomic probe hybridized also along the rest of the chromosomal regions. Our findings are in line with the general patterns observed in previous CGH-based reports (e.g. [66–69]) in the sense of biased hybridization in heterochromatic regions and point to the fact that even high amount of C0t-1 DNA is often insufficient to entirely outcompete highly repetitive (heterochromatic) regions (for related discussion, see [70]) (Figs 4 and 5).

### Genetic diversity analyses using the DArT-seq data

**Fig 6:**
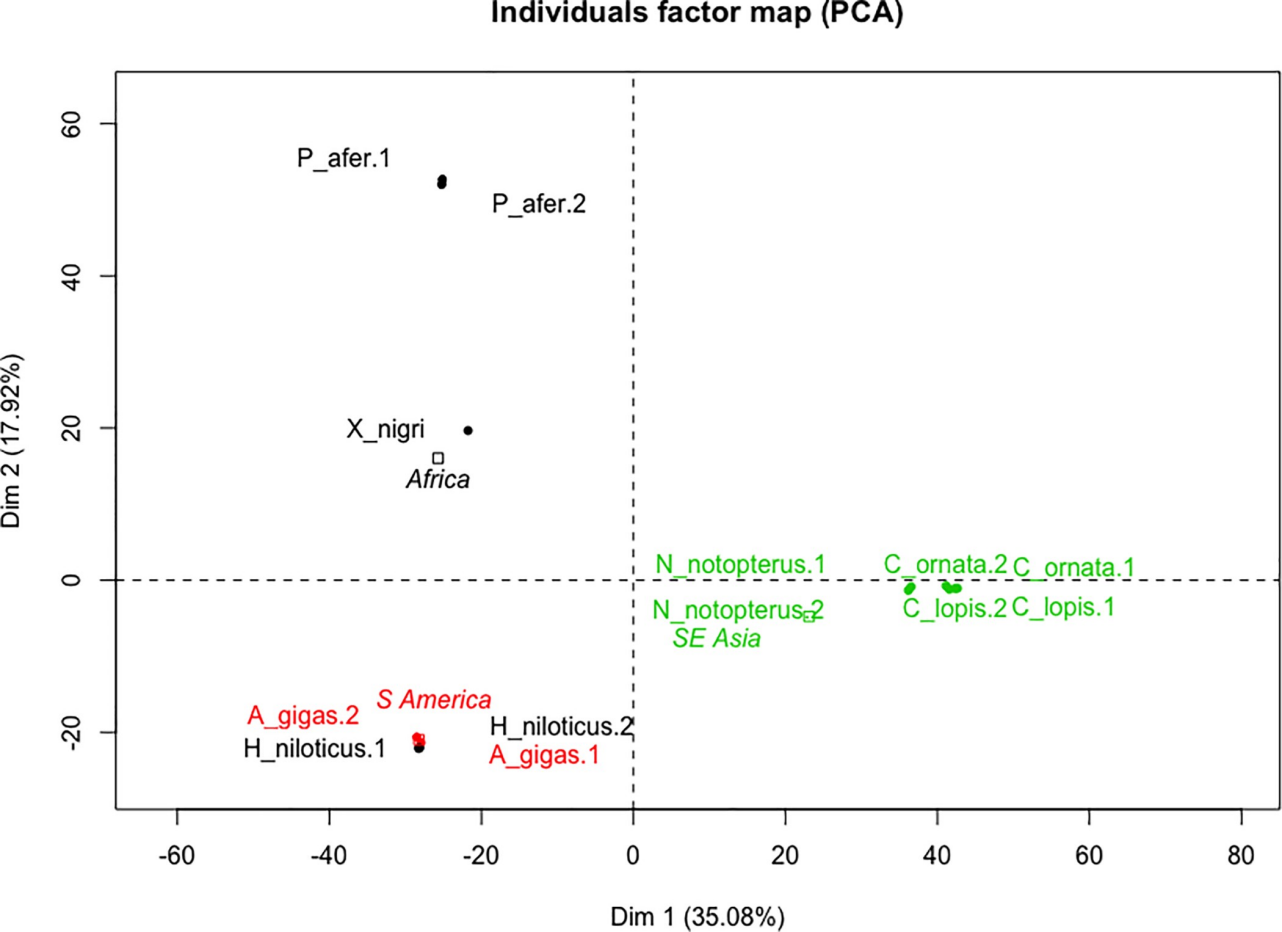
Principal component analyses (PCA) of SNP data in DArT-seq alleles found in seven osteoglossiform species. Individuals factor map using 3074 alleles (reference and alternative alleles). Osteoglossiform samples and presence/absence SNP were structured as observations (individual) and variable, respectively, as input matrix data. Notopteridae genera (*Papyrocranus*, *Xenomystus*, *Notopterus* and *Chitala*) and Arapamidae genera (*Heterotis* and *Arapaima*) together with their geographical distribution (Africa, Southeast Asia and South America); categorical variables are summarized in black (Africa), green (Southeast Asia) and red (South America) squares.

DArT-seq genotyping output **(S1 Table)** consisting of an “absence/presence” (0/1) matrix for each Arapaimidae and Notopteridae species (columns) for a given allele ID (rows), in which SNP calling relies on different statistical measures. An overview of the genotyping data showed that out of 1537 SNP alleles found, 57% showed transition type mutations, 88% presented only one SNP along the sequence and 19% were found in heterozygosity **(S1 Table)**. Principal component analyses using only the SNP alleles showed Notopteridae and Arapaimidae species clustered according to their geographical distribution. Partial results for Notopteridae were also discussed in Barby et al. [60] (Fig 6).

## Discussion

Osteoglossiformes represents one of the most ancestral, yet not well-studied, teleost lineages, and it is now widespread across all continents, except for Antarctica [71]. The lacking data are probably associated with the wide geographic distribution of this group, with taxa endemic to different continents, hampering an integrative study that would allow a globalized view of its accompanying evolutionary processes. Therefore, the present study focuses on the osteoglossiform family Arapaimidae, with aims to investigate unique fish model group using advanced molecular cytogenetic tools in an attempt to better explore and clarify drivers and certain events that have shaped its evolutionary and distribution history.

Cytogenetic data are still sporadic and quite incomplete for Osteoglossiformes in general, limiting the progress in understanding of the evolutionary trends operating in this group. Despite this, the data show that these fishes have diversified chromosome numbers, ranging from 34 in *Gymnarchus niloticus* to 56 in *A*. *gigas* and *Osteoglossum bicirrhosum* and karyotypes dominated by acrocentric chromosomes in most osteoglossiform species (reviewed by [35]). However, some representatives of Mormyridae, Gymnarchidae, and Notopteridae families share specific karyotype features, such as reduced 2n and a karyotype with bi-armed chromosomes, indicating a closer relationship among them (reviewed in [33]). On the other hand, while the majority of osteoglossiform species tend to maintain the karyotypes with acrocentric chromosomes, as stated before, the Arapaimidae and Gymnarchidae members represent exceptions to this general rule. Indeed, our data demonstrate 2n = 56 and a karyotype composed of 28 m/sm + 28 st/a chromosomes for *A*. *gigas* (FN = 84), agreeing well with some previous reports [34,35], but differing from Urushido [72]. In turn, *H*. *niloticus* displays 2n = 40 and a karyotype composed of 40 m/sm chromosomes (FN = 80) (Fig 2), also deviating from the single record previously published [73], who found the same diploid number (2n = 40) but inferred distinct karyotype composition (26m + 10sm + 4a chromosomes) for a West African population. The observed incongruences may have resulted from different morphological classification of some chromosomal pairs. Similar incongruences between karyotype studies are known also for osteoglossiform species *Pantodon buchholzi*, where the technical limitations in the former study were most likely responsible for improper karyotype characterization [33]. Unfortunately, as the locality of the *H*. *niloticus* specimens examined by [73] is not specified (since just “West Africa” is mentioned), we cannot exclude the possibility that these variations may also be related to some population variability.

**Fig 7:**
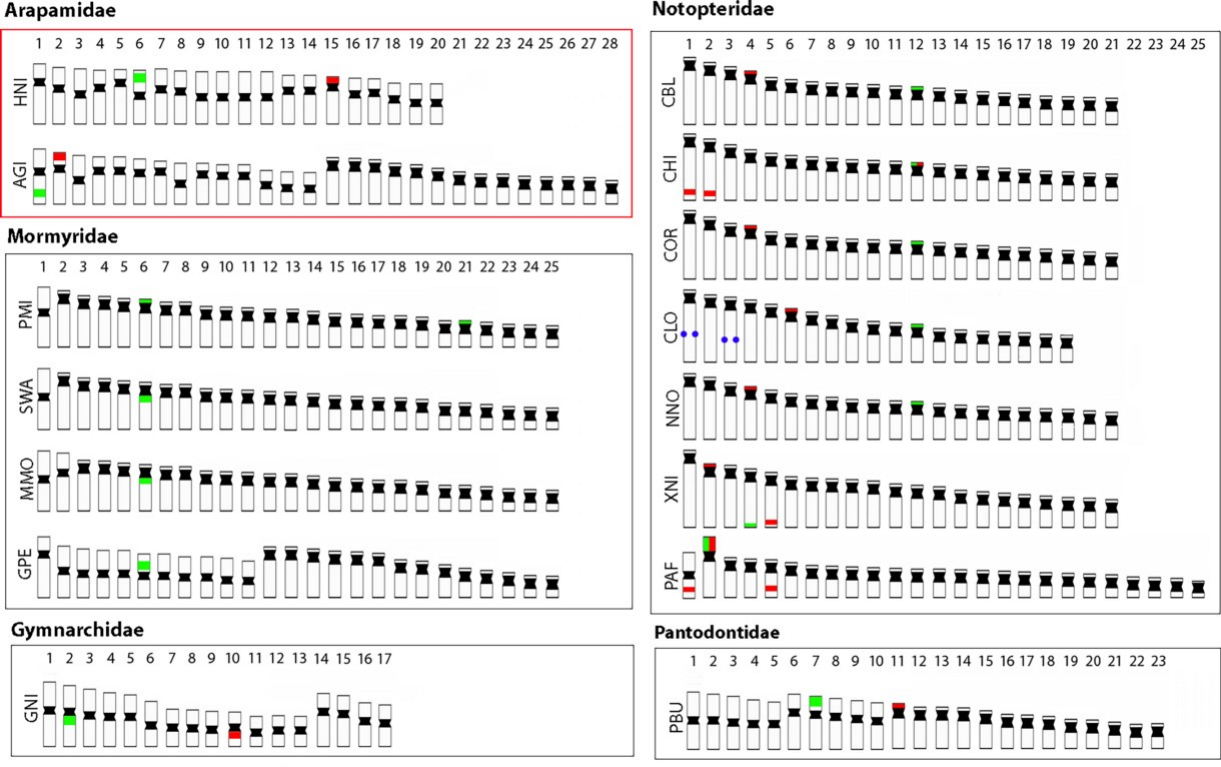
Idiograms representing 2n and patterns of rDNA distribution in osteoglossiform species. On the chromosomes, the distribution sites of 18S rDNA are highlighted in green, 5S rDNA in red, and Interstitial Telomeric Sites (ITS) in blue. HNI = *Heterotis niloticus*; AGI = *Arapaima gigas;* PMI = *Petrocephalus microphthalmus*; SWA = *Stomatorhinus walkeri;* MMO = *Marcusenius moorii;* GPE = *Gnathonemus petersii;* GNI = *Gymnachus niloticus*; CBL = *Chitala blanci;* CCH = *Chitala chitala*; COR = *Chitala ornate*; CLO = *Chitala lopis*; NNO = *Notopterus notopterus;* XNI = *Xenomystus nigri*; PAF = *Papyrocranus afer*; and PBU = *Pantodon buchholzi*. Data are based on [57,60,74].

In addition, the 18S rDNA probe identified a single locus in both species, with a very bright and hence arguably highly amplified signal in pair 15 of *H*. *niloticus* and in pair 2 of *A*. *gigas*, corresponding to the Ag-NOR sites in both species. In most of its analyzed species, osteoglossiform fishes possess only one chromosome pair bearing 18S and 5S rDNA sequences, with some exceptions, observed in *C*. *chitala*, *P*. *afer*, *X*. *nigri and Petrocephalus microphthalmus*) (for references and more detailed information about distribution of 18S and 5S rDNA sequences available to date in other osteoglossiform species, see Fig 7).

A single site (i.e. one pair of loci) for each rDNA cluster appears to be also the general trend reported for most teleosts [75,76] and ancient non-teleost actinopterygian fishes [77,78]. The location of such sequences in similar positions of similar chromosomes may be evidence of homeologous chromosomes (but see [79]). Among arapaimids, 18S rDNA sites are associated with GC-rich heterochromatin (Fig 3), a feature also shared with other osteoglossiform species, like the ones belonging to the Notopteridae and Gymnarchidae families [57,60], supporting the view that it likely represents the ancestral pattern for actinopterygian fishes [78]. However, besides a single merged 18S rDNA/ CMA_3_^+^ site _,_ multiple additional CMA_3_^+^ signals were also observed in both species, similarly to the pattern described in *P*. *buchholzi*, [33]. This pattern is otherwise very infrequent among teleosts (for references, see [80].

To gain deeper insight into gross-scale sub-chromosomal dynamics on the level of composition and distribution of repetitive DNA sequences, we performed CGH experiments in inter-specific comparative manner. These CGH comparisons showed overall high genomic divergence between the two species under study as might be expected due to their deep evolutionary divergence and corresponding advanced stage of repeat turnover (Figs 4 and 5). In fact, both genomic probes merged only in NOR sites which generally maintain high sequence conservativism [81]. In fact, when basically only the NOR regions are intensively labeled after cross-hybridization, it points to a large genomic divergence between both species. Such scenario has been also observed in several plants and other animal groups (e.g: [37,82]).

In summary, the genomes *A*. *gigas* and *H*. *niloticus* display remarkable chromosomal divergence, in terms of their karyotype macrostructure and repetitive DNA content and distribution. In striking contrast, the genomic diversity studies through PCA analyses demonstrated a very low genetic distance between these fishes on the level of unique/single copy regions (Fig 6). This scenario further deeply contrasts with the one found for African and Asian representatives of Old World knifefishes, family Notopteridae, where an opposite pattern was discovered, i.e., highly conserved karyotypes despite at least 80 My of isolation among the species [60]. Thus, what would have contributed to such enormous chromosomal diversity between Arapaimidae species?

An important hint may come from recently published genome of *Arapaima gigas* [83]. It is noteworthy that according to this study, only 21% of *Arapaima gigas* genome is composed of repetitive DNA, while other 33% represent coding regions. Similar proportion between repetitive DNA and coding sequences has been found also in the Asian arowana *Scleropages formosus* [84]. In the context of these genomic data and with respect to results yielded in this study, it might be imaginable that a strong selection might be operating in arapaimids to preserve sequence integrity of coding parts, which encompass substantial part of the genome. A possible outcome might be that repetitive sequences must accumulate in restricted genome areas and hence they might promote rearrangements in these specific locations due to elevated local dynamics (driven, for instance, by illegitimate recombination; e.g., [85]). This way, repetitive sequences may provide the fuel for karyotype variability, while coding regions retain high degree of sequence conservativity. The presence of elevated number of CMA+/GC-rich regions in both studied arapaimids may partly support our hypothesis as GC-rich regions, especially in conjunction with their terminal location on chromosomes, are more prone to high recombination rates (e.g., [86–88]). At the same time (or as an alternative explanation), higher flexibility of chromatin functional arrangement in interphase nuclei would be expected to be required to facilitate elevated plasticity for genome reshuffling and this flexibility might be, on the other hand, missing in Notopteridae fishes. This matter warrants further investigation especially in the context of current models of functional chromatin arrangement basing on topologically associating domains (TADs) [89–91].

Although separated by more than 50–80 Mya [5], *A*. *gigas* and *H*. *niloticus* still retain some similar morphological, physiological, and behavioral characteristics, such as obligatory branchial and aerial respiration, preference for low-oxygenated lentic environments, low migratory activities, and sophisticated parental care. Consequently, both species are sedentary, living in flocks with small population sizes and having a high degree of kinship and endogamy [10,92]. Molecular studies on individuals from the Amazon basin using microsatellite markers and mitochondrial genes have shown that different populations of *A*. *gigas* present some small degree of isolation by distance and fragmentation only in populations separated by distances greater than 1000 km [92,93]. In fact, higher karyotype variability is usually present in fish groups with low mobility and establishment of small isolated populations, in contrast with fish species characterized by higher mobility and population density [94–96]. In this context, chromosomal rearrangements spread and settle more easily in small populations, where the probability of generating homozygous rearranged forms, free of meiotic segregation problems, is greater than in large populations [97]. It is, however, worth mentioning that while the older theoretical models explained chromosomal speciation only in conjunction with geographic isolation in allopatric populations [98], current views enable to theoretize about this issue to happen also in sympatry, as the reproductive barriers may still evolve in the persisting presence of a gene flow [99]. This may happen if the strong selection acts to maintain linkage disequilibrium between locally adapted alleles via recombination arrest (through structural rearrangements and/or recombination modifiers), leading to gradual accumulation of sequence divergence only in a restricted genomic region [100]. In fact, the diversity of chromosomal types in *Arapaima* and *Heterotis* karyotypes is a result of several chromosomal rearrangements accumulated over millions of years, in addition to the accumulation of different classes of repetitive DNAs in their genomes. A similar scenario was already observed in another osteoglossiform species, the Asian Arowana *Scleropage formosus*, where repetitive DNAs are thought to be the major contributors to the chromosomal diversity observed in this species [84].

Our data support the view that highly rearranged karyotypes tend to occur in South American osteoglossiforms. *A*. *gigas* (2n = 56) and the South American arowana–*O*. *bicirrhosum* (2n = 56)–possess the highest 2n found in the order. This was also documented and verified in cichlid fishes, in which representatives from the Neotropical region display 2n = 48 chromosomes, with a greater karyotype diversity in comparison to the African ones [101,102]. Several fossil taxa were assigned to the Arapaimidae and/or closely related lineages (Fig 1), and, although their evolutionary interrelationships are not well established [9,24,103,104], we cannot exclude that the intermediate 2n numbers have occurred between them and the extant *A*. *gigas* and *H*. *niloticus*.

## Conclusions

Taken together, the genomes of *A*. *gigas* and *H*. *niloticus* displayed remarkable chromosomal divergence and repetitive DNA turn over. Our results demonstrated some general trends shared by most osteoglossiform species analyzed so far, like the presence of only one chromosome pair bearing 18S and 5S rDNA sites; karyotypes dominated by acrocentric chromosomes; and rDNA sites which are associated with GC-rich heterochromatin, supporting the view that it likely represents the ancestral state for teleost fishes. On the other hand, genomic diversity studied through PCA analyses demonstrated a very low genetic distance between these fishes despite separate evolutionary histories spanning approximately 50–80 My [5] and also the marked karyotype variability.

## Supporting information

S1 Appendix Detailed protocol for chromosomal obtainment in Arapaimidae fishes. (DOCX)

S1 Table List of all SNP data generated by DArTseq. FreqHomRef represents frequency of homozygotes for reference allele (more common major allele), FreqHomSnp represents frequency of homozygotes for SNP allele (less common minor allele), and FreqHets represents frequency of heterozygotes
(CSV)
