# Supplementary material for: Cytogenetics, genomics and biodiversity of the South American and African Arapaimidae fish family (Teleostei, Osteoglossiformes)
Source: PLoS One. 2019 Mar 25;14(3):e0214225. doi: 10.1371/journal.pone.0214225 (PMC6433368; doi:10.1371/journal.pone.0214225)
Supplement: S1 Appendix — (DOCX) [file pone.0214225.s001.docx]

**S1 Appendix: A detailed protocol for direct chromosome preparation from Arapaimidae fishes**

The direct preparations of chromosomes of arapaimid fishes were obtained following the protocol described by [1] with some modifications. Explicitly, considering that: i) the kidney is greatly enlarged in both *Arapaima gigas* and *Heterotis niloticus* and it may comprise a significant substratum for nitrogenous waste excretion and ii) there is evidence for the repositioning of physiological processes from the gills to the kidney in A. gigas, which is related to aerial respiration [2]; different organs (i.e., spleen, intestine, and gills) were selected as the source for the chromosomal preparation. Among them, the spleen showed the highest number of dividing cells, showing as the most suitable tissue.

Besides, in order to increase the mitotic index, the animals were stimulated by the procedures described in [3] with some modifications. A mixture containing 1ml of *Saccharomyces cerevisiae* suspension (12g of *S.cerevisiae* suspension + 12g of sucrose + 1ml of distillated water) per 100g body weight, for a period of 72h was injected intramuscularly. The treatment was repeated each 24h, in a sum of three injections. Anesthesia with a benzocaine solution (0,1%) was used to sacrify the fishes to minimize suffering followed by their immersion in an ice-slurry to achieve death by hypothermia. Portions of approximately 0,5 cm^3^ of the spleen were then removed and placed in a small cuvette containing 15ml of medium culture (RPMI 1640). Afterwards, small portions of the spleen were then fragmented using surgical tweezers and scissors followed by a delicate aspiration and expiration of the cells with a needle-free hypodermal syringe until a homogeneous cell suspension was obtained. 20µl of 0.025% colchicine solution was applied into the cell suspension, and again mixed and incubated at 36–37ºC in an incubator for 25 minutes.

The cell suspension was then centrifuged at 1000 rpm for 10 minutes, using a pipette the supernatant was discarded and suspended in 15 ml of hypotonic solution (0.075 M KCl) and maintained at 37 ° C for 60 minutes. Subsequently, 1.5 ml of freshly prepared Carnoy’s fixative (3:1 methanol/acetic acid) was added in the solution and the mixture was again centrifuged at 1000 rpm for 10 minutes. Finally, the supernatant was discarded with a Pasteur pipette and another 5–7ml of freshly prepared Carnoy’s fixative was added, letting it slide along the centrifuge tube walls. The tube were then again centrifuged at 1000 rpm for 10 minutes.

After the last centrifugation and supernatant elimination, 1 ml of freshly prepared Carnoy’s fixative was added, the solution was homogenized and the cell suspension were stored in an “Eppendorf” tube, and the slide preparations followed the air-drying methodology described in [1].

**References**

1. Bertollo LAC, Cioffi MB, Moreira-Filho O. Direct chromosome preparation from freshwater teleost fishes. In: Ozouf-Costaz C, Pisano E, Foresti F, Almeida Toledo LF, editors. Fish cytogenetic techniques (Chondrichthyans and Teleosts). CRC Press: Enfield USA; 2015. pp.21-26.
2. Brauner CJ, Matey J, Wilson N, Bernier J, Val AL. Transition in organ function during the evolution of air-breathing; insights from *Arapaima gigas,* an obligate air-breathing teleost from the Amazon. Journal of Experimental Biology. 2004; 207: 1433-1438.
3. Cavallini MM, Bertollo LAC. Indução de mitoses em *Hoplias* cf. *malabaricus* (Teleostei, Characiformes, Erithrinidae). Simposio de Citogenetica Evolutiva e Aplicada de Peixes Neotropicais. Maringa, Universidade Estadual de Maringa.; 1988.
